# Distal regeneration involves the age dependent activity of branchial sac stem cells in the ascidian *Ciona intestinalis*

**DOI:** 10.1002/reg2.26

**Published:** 2014-11-13

**Authors:** William R. Jeffery

**Affiliations:** ^1^ Eugene Bell Center for Regenerative Biology and Tissue EngineeringMarine Biological LaboratoryWoods HoleMA02543USA; ^2^ Department of BiologyUniversity of Maryland, College ParkMD20742USA

**Keywords:** aging, branchial sac, *Ciona intestinalis*, distal regeneration, stem cells

## Abstract

Tunicates have high capacities for regeneration but the underlying mechanisms and their relationship to life cycle progression are not well understood. Here we investigate the regeneration of distal structures in the ascidian tunicate *Ciona intestinalis*. Analysis of regenerative potential along the proximal−distal body axis indicated that distal organs, such as the siphons, their pigmented sensory organs, and the neural complex, could only be replaced from body fragments containing the branchial sac. Distal regeneration involves the formation of a blastema composed of cells that undergo cell proliferation prior to differentiation and cells that differentiate without cell proliferation. Both cell types originate in the branchial sac and appear in the blastema at different times after distal injury. Whereas the branchial sac stem cells are present in young animals, they are depleted in old animals that have lost their regeneration capacity. Thus *Ciona* adults contain a population of age‐related stem cells located in the branchial sac that are a source of precursors for distal body regeneration.

## Introduction

Regeneration is a central focus of current biological research because of its potential to provide solutions for the replacement of tissues and organs that are damaged or lost as a result of disease or injury. Mammalian organs, such as appendages, cardiac muscle, and spinal cord, have little if any regenerative capacities (reviewed by Poss 2010). With the exception of some amphibians and fishes, other vertebrates also show limitations in the scope of regeneration. In contrast, the invertebrate chordates, amphioxus and tunicates (including ascidians), have high regenerative capacities (Berrill 1951; Somorjai et al. 2012). The mechanisms of ascidian regeneration are important to understand because of the status of the tunicates as the closest living relatives of the vertebrates (Bourlat et al. 2006; Delsuc et al. 2006).

In the compound (colonial) ascidians *Botryllus and Botrylloides*, the entire body regenerates when zooids and developing buds are removed from a colony (reviewed by Tiozzo et al. 2008). Whole body regeneration is based on the activation of stem cells in the common basal vascular system (Rinkevitch et al. 1995; Tiozzo et al. 2008; Brown et al. 2009). Similarly, in the colonial ascidian *Clavellina*, each part of a bisected animal can regenerate the missing portion provided that it contains part of a coelomic pouch called the epicardium (reviewed by Berrill 1951). Presumably the epicardium is also a source of stem cells. In contrast to colonial ascidians, the solitary ascidian *Ciona intestinalis*, a model organism in developmental biology (Satoh 1994, 2014), has more restricted regeneration capacities. Classic experiments concluded that only the basal parts of bisected *Ciona* adults are able to regenerate the distal parts, whereas the original distal parts are unable to replace the basal parts (Hirschler 1914; see Jeffery 2014a for a review). Stem cells responsible for the regeneration of regional body parts have not been identified in *Ciona* or other solitary ascidians.

Recent studies of *Ciona* regeneration have focused on two organs located at the distal end of the body: the neural complex, which contains the cerebral ganglion or brain, and the oral siphon, a muscular tube leading into the pharynx. The oral siphon has orange‐pigmented sensory organs (OPOs) located in notches along its distal rim (Dilly & Wolken 1973). After their removal both organs are capable of complete regeneration from the basal portion of the body (Schultze 1899; Sutton 1953; Whittaker 1975; Bollner et al. 1992, 1993, 1995, 1997; Dahlberg et al. 2009; Auger et al. 2010). Studies of neural complex regeneration have centered on the cerebral ganglion, which takes about a month to replace, and includes healing of the overlying epidermis, the formation of a blastema of proliferating cells around the severed nerve endings, and the re‐growth and aggregation of neurons (Dahlberg et al. 2009). Oral siphon regeneration also involves blastema formation and is completed in about a month (Sutton 1953; Whittaker 1975; Auger et al. 2010). However, some oral siphon components, such as the siphon nerves and OPOs, are replaced more rapidly: in an average‐sized animal individual orange pigment cells differentiate in the siphon stump within 1−2 days, they aggregate into definitive OPO precursors by 4 days, and the OPOs are replaced by 6−8 days after amputation. The rapid replacement of OPOs during regeneration suggests an important physiological function, but their role during normal life and regeneration are unknown.

As is the case for many other animals (reviewed by Poss 2010), regeneration capacity declines as a function of age in *Ciona*, which live for about a year in nature and grow continuously before death (Berrill 1947; Millar 1952, 1953; Peterson et al. 1995). Regeneration of both the neural complex (Dahlberg et al. 2009) and the oral siphon (Auger et al. 2010) is affected by aging, although most current information on this relationship comes from studies of the oral siphon (Jeffery 2012). In young animals oral siphon and OPO regeneration are rapid, but their replacement becomes progressively slower during aging until it either ceases entirely or/and occurs in an aberrant form involving the formation of supernumerary or malformed OPOs (Jeffery 2012). Because of its short life cycle *Ciona* is being used as a model organism to study the mechanisms underling the reduction and loss of regeneration capacity during aging (reviewed by Jeffery 2014b).

Understanding the mechanisms of regeneration requires information about the source, mobilization, and function of progenitor cells. In many different animals, including the colonial ascidians, stem cells have essential roles in regeneration (reviewed by Tiozzo et al. 2008; Poss 2010). However, little is known about the identity and origin of the stem cells for neural complex, oral siphon, or OPO regeneration in *Ciona*. When the siphon is amputated near its tip, leaving a proximal stump, OPO replacement is controlled locally (Auger et al. 2010). Accordingly, explants of the amputated stump can regenerate complete OPOs after several days in culture without any other part of the body (Auger et al. 2010). When siphons are amputated at their base, however, OPOs are also regenerated, although this process requires more time, suggesting a distant source of progenitor cells.

This investigation was focused on the regeneration of distal structures in *Ciona*, primarily the oral siphon and its OPOs, with a major objective of determining the source of stem cells for this process. New regeneration experiments conducted here show that the branchial sac is a major source for stem cells involved in distal regeneration. Accordingly, stem cells located in the transverse vessels and lymph nodes of the branchial sac are described that provide progenitor cells to the distal regeneration blastema. It is also shown that these branchial sac stem cells are depleted during aging.

## Results

### Regeneration along the proximal−distal axis

To determine the regenerative capacity of different body parts, animals were bisected along a plane perpendicular to the proximal (basal)−distal axis (positions a−d; Fig. 1A). The separated parts were maintained in the original tunic to maximize survivorship and assessed for regeneration after a month in the laboratory culture system. The results indicated that basal body parts were able to regenerate the missing distal tissues, including the pharynx and branchial sac, neural complex, and both siphons with OPOs, but the distal portions were unable to regenerate any of the proximal parts and eventually disintegrated (Fig. 1B; Table 1). Confirming earlier studies (Hirschler 1914), even after the most basal bisection (position d, Fig. 1A), only the basal parts were able to regenerate complete animals. However, qualitative differences in regeneration were revealed depending on the position of the bisection. When animals were bisected distal to the exit of the rectum into the atrial cavity (position b, Fig. 1A) single oral and atrial siphons were regenerated (Fig. 1C), whereas when bisection was done proximal to this level (at positions c or d), animals regenerated a single oral siphon and either one (Fig. 1D) or two (Fig. 1E) atrial siphons (Table 1). The paired atrial siphons persisted for about 2 weeks and then gradually fused, producing a final regenerate with a single atrial siphon. During *Ciona* post‐metamorphic development, the atrial siphon is normally formed by fusion of two atrial siphon primordia (Chiba et al. 2004). Thus, distal regeneration from the basal parts recapitulates the original sequence of atrial siphon ontogeny.

**Figure 1:**
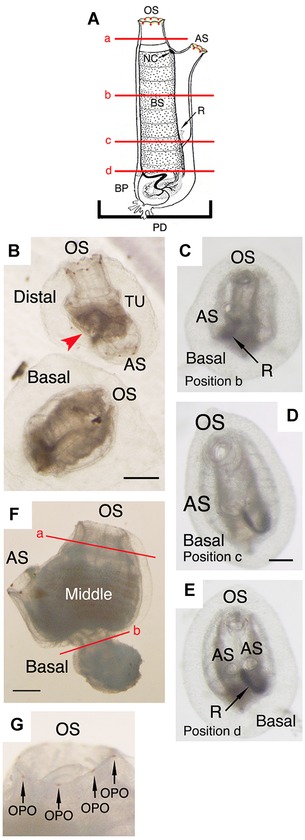
Regeneration of animals separated at different positions along the proximal−distal axis. (A) A diagram showing the approximate location of planes (horizontal red lines a−d) through which animals were separated into two or three parts. Distal is at the top and proximal (basal) is on the bottom. (B)−(E) Regeneration of animals cut into two parts along the proximal−distal axis. (B) Distal organs regenerate from the basal part, indicated by the re‐forming oral siphon (OS), but basal organs do not regenerate from the distal part, indicated by the red arrowhead at the cut surface. (C)−(E) Regeneration of an oral siphon (OS) and a single atrial siphon (AS) from the basal part of an animal bisected along plane b (C) or an oral siphon and one (D) or two (E) atrial siphons from the basal part of an animal bisected at plane c or d respectively. (F), (G) Regeneration of animals cut into three parts along the proximal−distal axis. (F) Regeneration of the oral siphon from the middle part of an animal severed along planes a and b (red diagonal lines). (G) 2× magnification of the regenerated oral siphon in (F) showing replaced pigmented sensory organs (OPOs). Scale bars in (B), (D), and (F) are 50 μm; magnification is the same in (C)−(E). TU, tunic; NC, neural complex; R, rectum opening into the atrial cavity; BS, branchial sac; BP, basal part containing the viscera. Regeneration experiments were performed on animals grown in the laboratory culture system from larvae attached to Petri dishes (PD).

To further investigate body regenerative capacity, animals were trisected by two successive cuts along the proximal−distal axis (positions a and b or c, Fig. 1A), producing distal, middle, and basal portions. The three parts were maintained within their original tunics for 10−14 days in culture and then examined to assess the extent of regeneration (Fig. 1F, G; Table 2). As described above, the most distal parts did not regenerate any of the proximal regions and eventually disintegrated. The basal parts showed the beginnings of distal regeneration (Table 2), as demonstrated in regenerating animals cultured for longer periods (Fig. 1B−E). However, the middle parts also regenerated distal parts, namely an oral siphon with OPOs (Fig. 1F, G; Table 1). The results show that middle portions of the body, which contain the branchial sac complex (Fig. 1A), have the potential to regenerate distal parts in the absence of basal parts.

**Table 1:** Regeneration of animals cut into two parts.

Position of	Total	Distal part	Basal part	One	Two
operation^a^	animals^b^	regeneration	regeneration	atrial siphon	atrial siphons
a	26	0	25	—	—
b	35	0	30	30	0
c	27	0	19	10	9
d	35	0	15	2	13

^a^See Figure 1A. ^b^Animal parts indicated as not regenerated disintegrated during culture.

**Table 2:** Regeneration of animals cut into three parts.

Position of	Total	Distal part	Middle part	Basal part
operations^a^	animals^b^	regeneration	regeneration	regeneration
a and b	34	0	22	22
a and c	16	0	11	11

^a^See Figure 1A. ^b^Animal parts indicated as not regenerated disintegrated during culture.

### Role of cell proliferation in distal regeneration

A blastema of proliferating cells is formed at the site of distal regeneration beginning about 4 days after oral siphon amputation (Auger et al. 2010). To investigate the role of cell proliferation in OPO regeneration, the effects of mitotic inhibitors and labeling with the cell proliferation marker EdU on OPO replacement were determined. Animals were pre‐incubated with colchicine (*N* = 15) or nocodazole (*N* = 8) for 10 h and then bisected at site a (Fig. 1A). After bisection, the basal parts were incubated with EdU for 2 days and chased for 4 (position a) or 8 (position b) days. The ability to replace the OPOs in animals was not affected by drug treatment (Fig. 2A−D), although the drugs reduced EdU incorporation substantially compared with controls (see for example nocodazole in Fig. 2C, D). In EdU labeling studies carried out after bisection at position b, OPOs, as well as the orange pigment spot on the dorsal side of the cerebral ganglion, regenerated without EdU labeling (Fig. 2E, F). Thus, although cell proliferation appears to occur throughout the blastema, it is not required for the differentiation of individual orange pigment cells, OPOs, and other pigmented organs.

**Figure 2:**
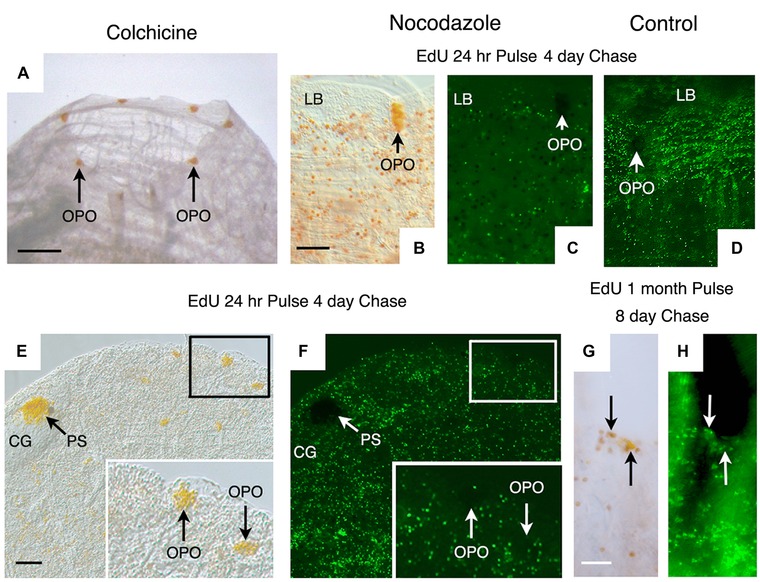
Role of cell proliferation in distal regeneration. (A)−(C) Pigmented sensory organs (OPOs) regenerate in the basal portions of colchicine (A) or nocodazole (B)−(D) treated animals after bisection at position a (Fig. 1A). (A) Regenerated OPOs in colchicine treated animals. Scale bar 2 mm. (B)−(D) Regenerated unlabeled OPOs in a nocodozole and EdU treated animal. (B) Bright field image of (C). (C), (D) Fluorescence images of nocodozole treated (C) and untreated control (D) animals labeled with EdU for 24 h and chased for 4 days after bisection. LB, oral siphon lobe. (E), (F) Unlabeled OPOs and other orange pigmented organs regenerate in the basal portion of an animal labeled with EdU for 24 h and chased for 4 days after bisection at position b (Fig. 1A). PS, pigment spot covering the cerebral ganglion (CG). Boxes are insets of approximately 2× magnification in (E), (F). Scale bars in (B) and (E) are 20 μm; magnification is the same in (B)−(D) and in (E), (F). (G), (H) Regenerated EdU labeled OPOs beginning to form in an animal labeled with EdU for 1 month, then bisected though plane b (Fig. 1A), and chased for 8 days. Arrows, regenerating OPOs. Scale bar 40 μm; magnification is the same in (G) and (H). (A), (B), (E), and (G) are bright field images. (C), (D), (F), and (H) are fluorescence images.

To determine whether dividing cells could be detected in regenerating OPOs after longer periods of EdU labeling, animals (*N* = 6) were pre‐incubated with EdU for 1 month, and then were cut at position b (Fig. 1A) and the basal parts were chased for 10 days. In all of these animals orange pigment cells in the forming OPOs showed EdU labeling (Fig. 2F, G). Therefore, in contrast to most cells involved in distal regeneration, the progenitors of orange‐pigmented organs are derived from cells that may be slowly replenished during the life cycle.

### Source of stem cells for distal regeneration

Two approaches were used to determine the source of stem cells for distal regeneration. In the first approach, it was reasoned that dividing cells involved in regeneration might be detectable by their early cell proliferation in regenerating but not control animals. Thus, to determine the location of early dividing cells, animals were bisected at site a (Fig. 1A) and exposed to EdU for 3, 8, 24, or 48 h, and the location of EdU labeled cells was compared in control (unoperated) and regenerating animals. Control animals showed high levels of EdU labeling in the stomach, intestines, and basal stalk, but negligible labeling in other locations of the body (Fig. 3F). It is well known that stomach and intestinal cells are subject to continuous turnover and replacement in *Ciona* and other ascidians (Ermak 1975a, 1976a, 1981). Control and regenerating animals showed about the same levels of EdU labeling in the visceral organs (Fig. 1A−F), suggesting that there were no changes elicited by distal regeneration. The visceral tissues also could be excluded from participation in distal regeneration because middle body parts lacking them were able to regenerate distal structures (see above, Fig. 1E). Apart from the viscera, the only other tissue showing early EdU incorporation in regenerating animals was the transverse vessels of the branchial sac (Fig. 3A−D), which were strongly labeled even after the first 3 h of regeneration, and their intense labeling continued throughout the experiment (Fig. 3A−D). EdU labeling was most prominent in the lymph nodes (Fig. 3G, H), which are spaced periodically along the transverse vessels and known to be involved in blood cell renewal (Ermak 1975b). The regenerating oral siphon and the endostyle, which has been proposed as a stem cell niche in colonial ascidians (Voskoboynik et al. 2008; Rinkevich et al. 2013), did not show appreciable EdU labeling until 48 h after amputation, and the atrial siphon always had negligible levels of EdU incorporation (Fig. 3A−E). In contrast to the basal parts of the bisected animals, the distal parts, which included the excised oral siphon, did not show appreciable levels of EdU incorporation with the exception of the tentacles (see Fig. 3J).

**Figure 3:**
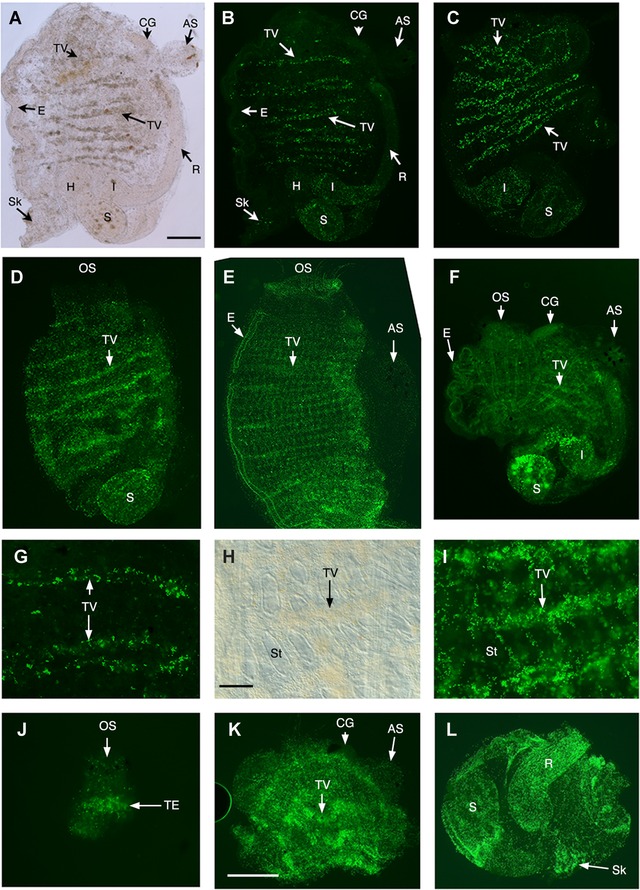
Early cell proliferation during distal regeneration. (A)−(E), (G)−(I) EdU labeling of the basal regenerating parts of animals for 3 h (A, B), 12 h (C), 24 h (D), or 48 h (E) after bisection through position a (Fig. 1A) shows early labeling in the transverse vessels of the branchial sac. (G)−(I) Images of animals in (B) and (D) magnified about 2× show labeling in transverse vessels (TV) of the branchial sac. (F) Non‐regenerating control labeled with EdU for 24 h showing labeling primarily in the viscera. (J)−(L) Parts of animals trisected at positions a and b (Fig. 1A) and then incubated with EdU for 48 h showing the distal (J), middle (K), and basal (L) parts. (A), (H) Bright field images of (B) and (I) respectively. (B)−(G), (I)−(L) Fluorescence images. OS, oral siphon; CG, cerebral ganglion; AS, atrial siphon; TV, transverse vessels; E, endostyle; H, heart; R, rectum; I, intestine; S, stomach; Sk, basal stalk; St, stigmata; TE, oral tentacles. Scale bar in (A) is 60 μm; magnification is the same in (A)−(F) and (J). Scale bar in (H) is 15 μm; magnification is the same in (G)−(I). Scale bar in (K) is 100 μm; magnification is the same in (K) and (L).

Similar results were obtained for animals cut into three parts (at sites a and b; Fig. 1A): in the basal portion strong EdU labeling was restricted to the viscera and transverse vessels of the branchial sac, in the middle portion EdU labeling was seen in the transverse vessels of the branchial sac, and the distal portion showed negligible labeling except for the tentacles (Fig. 3J−L). As separation at position a was approximate, the ring of tentacles was sometimes included or excluded from the distal portion of the trisected animal, but there was no consequence on EdU labeling levels in the body wall of the distal fragments. The results indicate that the initial surge of cell division relevant to distal regeneration is restricted to the transverse vessels and lymph nodes of the branchial sac.

In the second approach, the location of stem cells was investigated using alkaline phosphatase (AP) and PIWI markers. AP has been used as a pluripotent stem cell marker in vertebrates (Riekstina et al. 2009) and colonial ascidians (Akhmadieva et al. 2007). As shown in Figure 4A, AP staining was observed in the stomach, intestine, rectum, and basal stalk. However, the most intense AP activity was seen in transverse vessels of the branchial sac (Fig. 4A, B). AP staining in the transverse vessels was reduced after animals were pre‐treated with the AP inhibitor levamisole (Fig. 4B, C). Small foci of AP stained cells were also scattered in the body wall of the oral siphon, although the OPOs showed no staining (Fig. 4D, E).

**Figure 4:**
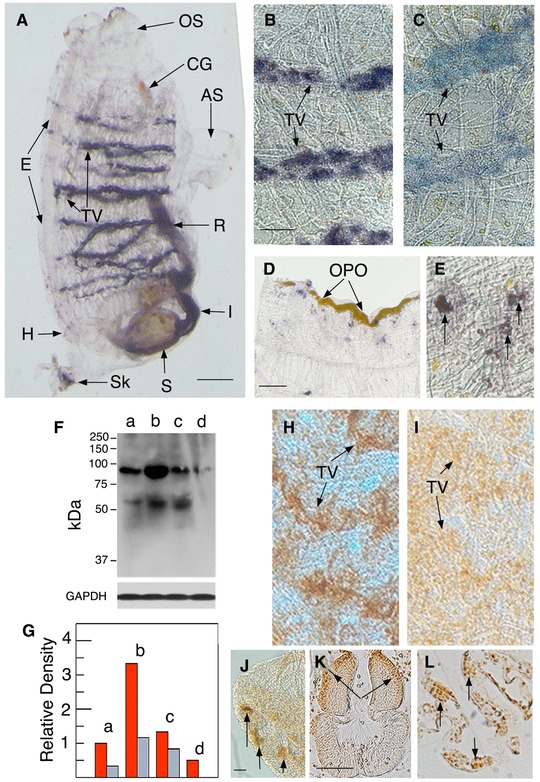
Stem cells detected by AP and PIWI markers. (A)−(E) AP stained stem cells. (A) An adult showing AP staining in the transverse vessels (T) of the branchial sac, rectum (R), intestine (I), stomach (S), and basal stalk (Sk). OS, oral siphon; CG, cerebral ganglion; AS, atrial siphon; E, endostyle. Scale bar 50 μm. (B), (C) AP staining in the transverse vessels in the absence (B) or presence (C) of levamisole. Scale bar in (B) is 25 μm; magnification is the same in (B), (C), (H), (I). (D) Flat mount of an oral siphon showing AP staining in small regions below and adjacent to the oral siphon pigment organs (OPOs). (E) 4× magnification of (D) shows small clusters of AP stained cells in the oral siphon. Animals were turned inside out prior to AP staining (see Materials and Methods). (F)−(L) PIWI stained stem cells. (F) Western blot (upper frame) showing 95 and 56 kDa bands detected by PIWI antibody in extracts of (a) a 6‐month‐old whole animal, (b) an isolated gonad from a 6‐month‐old animal, (c) an isolated branchial sac from a 6‐month‐old animal, and (d) an isolated branchial sac from a 14‐month‐old animal. GAPDH loading standard (lower frame). (G) Semi‐quantitative analysis of 95 kDa band (red bars) and 56 kDa band (grey bars) densities in (F). (H)−(L) Immunostaining showing PIWI expression in the branchial sac (H), basal stalk (J), endostyle (K), and transverse vessels (L). (I) Immunostaining with a control (OCA2) antibody shows background staining in the branchial sac. (K) and (L) are sections. Scale bar in (J) is 30 μm. Scale bar in (K) is 10 μm; magnification is the same in (K) and (L).

PIWI, an Argonaute family protein involved in maintaining stemness, has also been used as a stem cell marker in many animals (Cox et al. 1998; Seipel et al., 2004; Palalkodeti et al. 2008), including colonial ascidians (Brown et al. 2009; Rosner et al. 2009; Rinkevich et al. 2010). The *Ciona intestinalis* genome contains two *piwi* orthologs (Satou et al. 2001, 2002). A polyclonal antibody was used against a highly conserved domain in *Drosophila* PIWI to detect PIWI protein in *Ciona*. The PIWI antibody detected major bands of about 95 and 56 kDa on western blots (Fig. 4F, G), as reported previously in the ascidian *Botrylloides violaceus* (Brown et al. 2009). The 95 kDa band is about the expected molecular mass of the *Ciona* PIWI like‐2 protein, and the lower molecular mass band could be another PIWI ortholog or a breakdown product. Western blots showed the same bands in protein extracts from 3‐month‐old whole animals (Fig. 4Fa), isolated branchial sacs (Fig. 4Fb), and isolated gonads (Fig. 4Fc). The presence of PIWI in gonads confirms its expression in germ‐line tissues (Sunanaga et al. 2010; Kawamura and Sunanaga 2011). Immunostaining showed PIWI expression in the basal stalk (Fig. 4J), the endostyle (Fig. 4K), and in cells within transverse vessels of the branchial sac (Fig 4H,L), which are tissues that also exhibit PIWI expression in colonial ascidians (Brown et al. 2009; Rinkevich et al. 2010; Sunanaga et al. 2010). Immunostaining with a control antibody did not label these tissues (Fig. 4I), supporting the specificity of PIWI detection. The results show that transverse vessels of the branchial sac contain AP and PIWI expressing stem cells in *Ciona*.

Together the EdU labeling and stem cell marker results suggest that the transverse vessels of the branchial sac are a potential source of stem cells for distal regeneration.

### Relationship between stem cells and dividing cells

AP‐EdU double‐labeling experiments were performed to determine the relationship between stem cells and proliferating cells in the branchial sac of regenerating animals. In these experiments, animals bisected at position a (Fig. 1A) were labeled with EdU for 24 h and then processed immediately for AP staining. The results showed co‐localization of EdU labeling and AP expression in the transverse vessels after the 24 h EdU pulse (Fig. 5A, B). Sectioning of these double‐labeled animals demonstrated three kinds of cells in the transverse vessels/lymph nodes with regard to AP and EdU staining: some cells expressed AP but lacked EdU labeling, some cells showed only EdU labeling, and other cells showed both AP expression and EdU labeling (Fig. 5C, D). To determine the relationship between these cells, AP stained and EdU labeled cells were quantified in animals subjected to a 24 h EdU pulse or a 24 h EdU pulse and a 4 day chase prior to AP staining (Fig. 5E). The number of AP stained cells was not significantly different after the pulse or the pulse‐chase experiment. The numbers of EdU labeled cells appeared to increase, although the increase was not significant. However, the number of double‐labeled cells significantly decreased during the chase. These results suggest that some of the AP stained stem cells are precursors of proliferating cells in the branchial sac.

**Figure 5:**
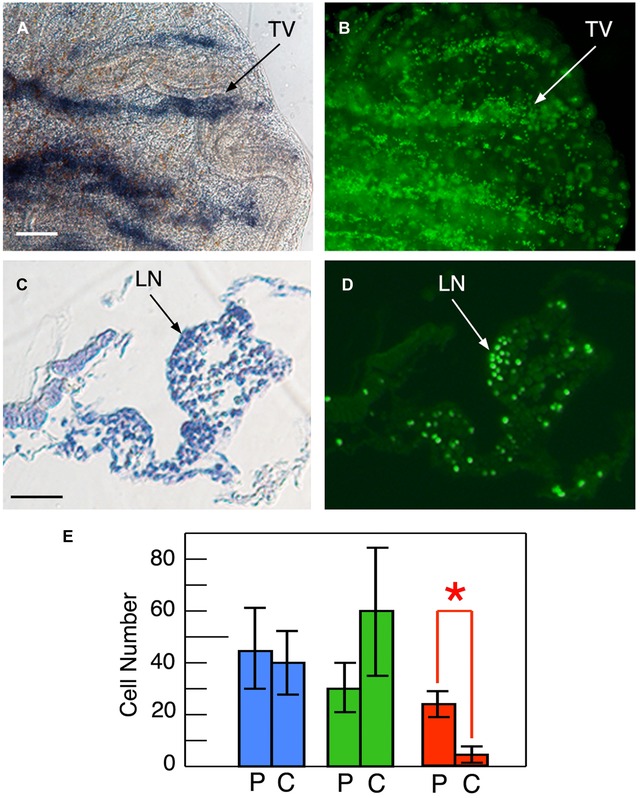
The relationship between AP stained stem cells and proliferating cells during distal regeneration. Fluorescence (B, D) and bright field (A, C) images of whole mounts (A, B) and sections (C, D) of the basal portions of regenerating animals that were pulse labeled with EdU for 24 h, then bisected at position a (Fig. 1A), and stained with AP. TV, transverse vessels; LN, lymph node. Scale bar in (A) is 50 μm; magnification is the same in (A) and (B). Scale bar in (C) is 10 μm; magnification is the same in (C) and (D). (E) The relationship between AP stained, EdU labeled, and AP/EdU double‐labeled cells. Animals were labeled with EdU for 24 h, bisected at position a (Fig. 1A), and then either immediately processed for AP staining (pulse, P) or chased for 4 days and then stained with AP (chase, C). The blue bars indicate AP stained cells, the green bars EdU labeled cells, and the red bars AP/EdU double‐labeled cells. Each bar represents the mean number of cells ± SD. Cells were counted in 100 μm^2^ regions of three consecutive serial sections in the middle of the branchial sac of eight different animals. Statistical analysis was conducted with Student's *t* test. *P > 0.001.

### The branchial sac is a source of stem cells in the distal regeneration blastema

Several different approaches were used to determine the source of dividing and non‐dividing cells in the distal regeneration blastema. Initial experiments used an EdU pulse‐chase protocol to determine if cells labeled in the branchial sac could be chased into the blastema. Control animals and the basal parts of regenerating animals bisected at position a or b (Fig. 1A) were exposed to EdU for 24 h and then chased without EdU for 5−10 days. At the end of the chase, these controls showed the usual labeling in the stomach and intestine, lower levels of labeling in the branchial sac, and no appreciable labeling in the siphons (Fig. 6A, B). In contrast, regenerating animals had labeling in the viscera and branchial sac but also showed intense labeling in the distal regeneration blastema (Fig. 6C, D), which was not labeled at the end of a 2 day EdU pulse (Fig. 3D), suggesting a source of proliferating cells outside the blastema.

**Figure 6:**
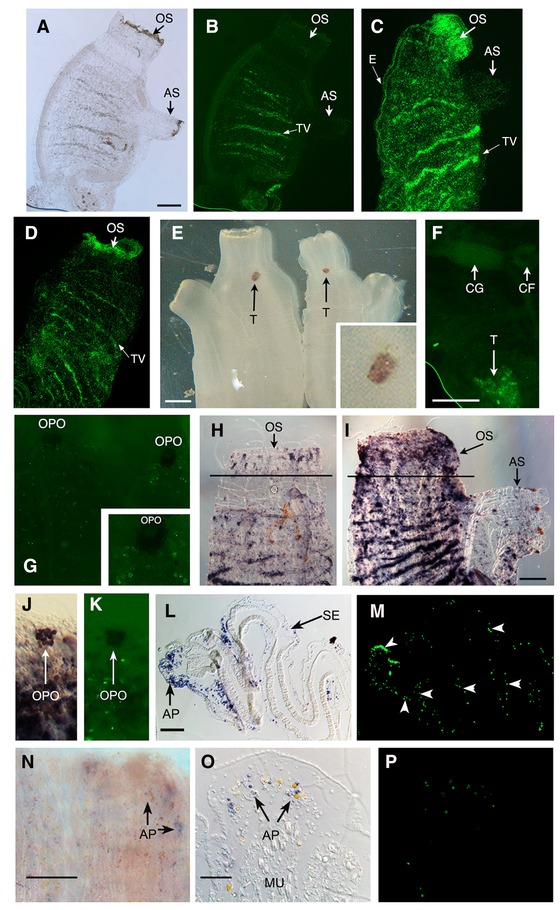
Role of the branchial sac in distal regeneration. (A)−(D) Images of control (A, B) and regenerating (C, D) animals following a 48 h EdU pulse and 4 day chase. (A), (B) Bright field (A) and fluorescence (B) images of an unoperated control animal. (C), (D) Fluorescence images of the basal parts of animals separated at position a (C) or b (D) (Fig. 1A). OS, oral siphon; AS, atrial siphon; E, endostyle; TV, transverse vessel. Scale bar in (A) is 60 μm; magnification is the same in (A)−(D). (E)−(G) Branchial sac transplantation experiments. (E) Hosts with neutral red stained donor branchial sac transplants (T). Scale bar is 3 mm. Inset: the left transplant in (E) magnified about 3×. (F) The mostly unlabeled region between the EdU labeled transplant (T) and the cerebral ganglion (CG) of a host. CF, ciliated funnel. Scale bar 100 μm. (G) The oral siphon of a host with EdU labeled cells. Scale bar 50 μm. Inset: 2× magnification of (G) showing EdU labeled cells around the oral siphon pigmented organs (OPOs) in the regenerated host. (H)−(M) AP staining in distal regenerating tissues of animals bisected at position a (Fig. 1A). (H), (I) Three (H) and 5 (I) days post‐operation. (J), (K) Bright field (J) and fluorescence (K) images of AP stained and unlabeled OPOs in the 3 day regenerating oral siphon. Scale bar in (I) is 40 μm; (H) and (I) are the same magnification. (J) and (K) are 5× magnifications of (I). (L), (M) Bright field (L) and fluorescence (M) images of an AP‐EdU double‐stained oral siphon sectioned through a horizontal plane 5 days post‐operation. SE, siphon epidermis; AP, AP stained cells; arrowheads, EdU labeled cells. Scale bar in (L) is 100 μm; (L) and (M) are the same magnification. White arrowheads in (M) show EdU labeled cells. In (H), (K) animals were turned inside out (see Materials and Methods) prior to AP staining. (N), (O) Oral siphon explants doubled labeled with AP and EdU for 5 days. (N) Explant whole mount. (O), (P) Bright field (O) and fluorescence (P) images of the same explant section. MU, muscle fibers. Scale bar in (N) is 40 μm. Scale bar in (O) is 100 μm; magnification is the same in (O) and (P).

A transplantation approach was developed to test whether proliferating cells in the branchial sac could migrate into distal regenerating tissues and participate in blastema formation. In this procedure, the basal portions of small animals bisected at position a (Fig. 1A) were given a 48 h EdU pulse as described above; then the labeled branchial sacs were removed, vitally stained with neutral red, and transplanted into the distal branchial sac of larger host animals (Fig. 6E). Regeneration was induced in the host animals by bisection at position a (Fig. 1A), leaving the transplanted tissue in the basal part. After 10−15 days, EdU labeled cells were detected in host tissues lying distal to the transplant, including the neural complex and most prominently the regenerating oral siphon (Fig. 6F, G; Table 3). Similar to previous results (Fig. 2A−E), EdU labeling was not detected in the newly formed OPOs of the regeneration blastema, and labeling was low in host tissues proximal to the donor transplant (Table 3). Controls that received a labeled branchial sac transplant but were not induced to regenerate also showed no appreciable EdU labeling in their distal regions (Table 3). These results suggest that proliferating cells from the branchial sac migrate into the blastema during distal regeneration.

**Table 3:** Levels of proliferating cells in distal regenerating and proximal non‐regenerating tissues after transplantation of branchial sacs from EdU labeled donors into unlabeled hosts.

Animal		Number of distal EdU	Number of proximal EdU
type	*N*	labeled cells ± SD^a^	labeled cells ± SD
Transplant/regenerating	18	16.1 ± 9.5^b^	1.39 ± 2.8^c^
Transplant/non‐regenerating	5	1.4 ± 1.67^d^	3.0 ± 2.74
Control (no transplant)	3	0	0

^a^Mean numbers of EdU labeled cells counted in 10 randomly selected 200 μm^2^ regions of flat mount preparations (Auger et al. 2010) containing the oral siphon (for distal) and near the base of the branchial sac (for proximal) of hosts. ^b^ *P* = 0.0001 with respect to c and d.

The approaches described above did not address the origin of non‐dividing cells in the regeneration blastema, including those that produce the new OPOs. Therefore, additional regeneration experiments were done to determine whether changes in AP stained cells themselves occur during distal regeneration. In these experiments, animals were bisected at position a (Fig. 1A), and the distal and basal parts were cultured for several days prior to AP staining. The number of AP stained cells did not change in the non‐regenerating distal fragments; however, the regenerating distal areas in the basal fragments showed localized masses of AP stained cells after 3 (Fig. 6H) or 5 (Fig. 6I, J, L) days of regeneration. After 5 days of regeneration, strong AP staining was also detected in regenerating OPOs (Fig. 6J) but no EdU labeling was evident in the OPOs (Fig. 6K, M) or other AP stained cells in the blastema (Fig. 6L, M).

The increase in AP stained cells in the regeneration blastema could be caused either by new AP expression in cells already present at the ablation site or by the invasion of stem cells from the branchial sac. To distinguish between these possibilities, regenerating oral siphon explants, which lack proximal parts of the body (Auger et al. 2010), were cultured *in vitro* (Fig. 6N−P). After 5 days of culture, these explants contained only the small foci of AP stained cells that are typical of the unoperated oral siphon (Fig. 4D, E) and not the massive regions of AP staining cells normally seen in regeneration blastema (Fig. 6L). Furthermore, very few EdU labeled cells were detected in these explants (Fig. 6P). These results suggest that the non‐dividing cells of the blastema originate as AP stained stem cells in the branchial sac, which invade the distal areas and differentiate into OPOs early during regeneration.

### Branchial sac stem cells are depleted in old animals

It was next asked whether the reduced capacity for distal regeneration during aging (Auger et al. 2010; Jeffery 2012) is related to changes of stem cells in the branchial sac. In contrast to previous studies in which size was used as a proxy for age in wild collected animals (Jeffery 2012), the present studies employed animals of known ages cultured in the laboratory from fertilized eggs: 6‐month‐old animals (young animals) were compared with 12‐month‐old animals (old animals). The old animals were distinguishable from young animals in having thicker tunics, OPO malformations on the rims of their oral siphons (Jeffery 2012), and the absence of gametes in their gonoducts. The basal parts of young animals bisected at position a (Fig. 1A) showed normal distal regeneration (*N* = 6; Fig. 7A). In contrast, old animals (*N* = 14; Fig. 7B) were either unable to regenerate (12 animals) or regenerated partial siphons (two animals) within a 14 day period. Both young and old animals showed proliferating cells in the branchial sac (Fig. 7C, D) but the distribution of these cells, as well as the structure of the branchial sacs, appeared to be disorganized in old animals (Fig. 7D). Young animals showed strong AP activity in transverse vessels (Fig. 7E), similar to that described above for 3‐month‐old animals (Fig. 4A, B). In contrast, the non‐regenerating old animals showed weak, if any, AP stained cells in transverse vessels (Fig. 7G). The few old animals with residual regeneration capacity had higher levels of AP staining in parts of their branchial sacs (Fig. 7F), but this was not comparable with that in young animals (Fig. 7E). The AP staining results were confirmed by assaying PIWI expression in young and old animals. The results showed that the 95 kDa and 56 kDa PIWI bands were decreased in the isolated branchial sacs (Fig. 4Fc, d) and PIWI staining was reduced in the transverse vessels of old versus young animals (Fig. 7H, I). The results suggest that AP and PIWI stained stem cells are depleted in the branchial sac during aging.

**Figure 7:**
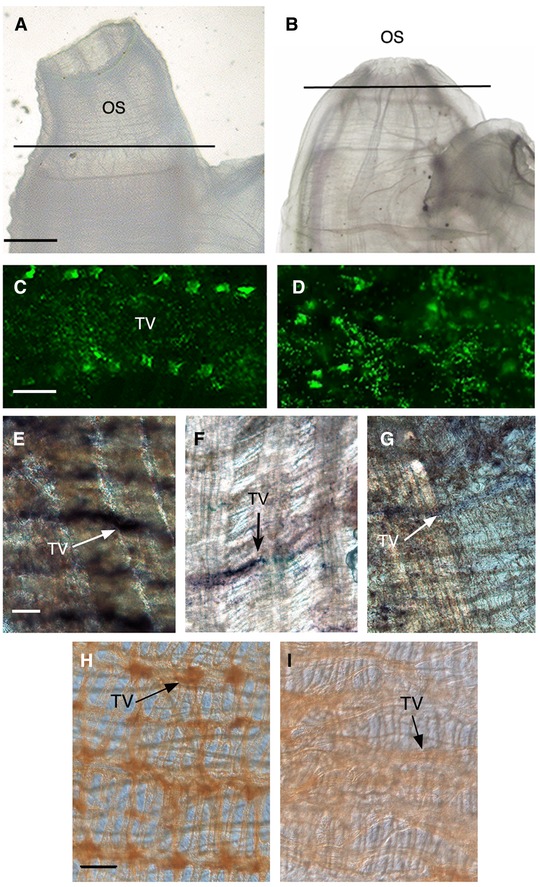
Effects of aging on distal regeneration, cell proliferation, and branchial sac stem cells. (A), (B) Distal regeneration of the oral siphon in the basal part of a young (A) but not a typical old (B) animal. The position of bisection was in the region between the base of the oral siphon and the hood of the endostyle (shown by horizontal black lines). Scale bar in (B) is 4 mm; magnification is the same in (A) and (B). (C), (D) Cell division in the branchial sacs of regenerating young (C) and old animals labeled for 2 days with EdU. TV, transverse vessel. (E)−(G) AP staining of stem cells in the transverse vessels (TV) of young and old animals. (E) Young animal. (F) Old animal with residual regeneration capacity. (G) Old animal with negligible regeneration capacity. (H), (I) PIWI staining of stem cells in the TV of young (H) and old (I) animals. Scale bar in (C) is 80 μm; magnification is the same in (C), (D). Scale bar in (E) and (H) is 10 μm; magnification is the same in (E)−(I).

## Discussion

Ascidians have extensive capacities for regeneration but the underlying mechanisms are not well understood. The present investigation has revealed some key principles involved in distal regeneration of the solitary ascidian *Ciona intestinalis*. This study has shown that a class of age‐related stem cells located in the branchial sac of *Ciona* adults provide precursors for the regeneration of distal tissues and organs.

### Distal regenerative capacity

Classical studies (see Jeffery 2014a for a review) showed that distal parts of bisected *Ciona* adults could regenerate from the proximal (basal) parts of the animal. However, the distal parts were unable to replace any of the basal tissues and organs. These results have been confirmed and extended here to reveal two new findings concerning *Ciona* regeneration. First, distal regeneration from the basal body recapitulates the original mode of adult body development: the replacement of an atrial siphon begins with the formation of two siphon primordia that later fuse into a single atrial siphon. The same process occurs during post‐metamorphic atrial siphon morphogenesis (Chiba et al. 2004), and this suggests that distal regeneration involves a reversion to the original program involved in adult development. Second, after the adult body is separated into three parts, the centrally located body fragment, which contains the branchial sac complex, can regenerate distal structures, namely a new oral siphon and associated tissues and organs, without the participation of the basal portion of the animal. This requires a revision of the hypothesis that distal regeneration is controlled by the most basal part of the animal (Hirschler 1914). Instead, the current data imply that distal regeneration requires a part of the animal containing the branchial sac complex. Consistent with this idea, Hirschler (1914) did note that distal regeneration occurred from basal parts only if they contained a piece of the branchial sac. The new hypothesis does not change the conclusion that distal parts of the body cannot regenerate basal parts, which has been confirmed in our study. However, the results focus new attention on the role of the branchial sac complex in regeneration of the oral siphon and other distal structures.

### Contrasting cell types in the regeneration blastema

Previous studies showed that distal regeneration involves proliferating cells that differentiate in a blastema at the wound site (Dahlberg et al. 2009; Auger et al. 2010). The present studies revealed that this blastema contains at least two different types of progenitor cells distinguishable by whether or not they participate in cell division and their timing of appearance in the blastema during regeneration (Fig. 8). First, a subset of branchial sac cells shows incorporation of EdU into DNA early during distal regeneration, but there is a delay of several days before the labeled cells are detectable in the blastema (see below). Second, another subset of branchial sac cells invade the blastema very soon after the initiation of regeneration and differentiate into the OPOs without undergoing cell division. Both types of branchial sac stem cells presumably interact with body wall epidermis to regenerate distal tissues and organs. The contribution of dividing and non‐dividing cells to the blastema suggests that multiple types of progenitor cells function in parallel during *Ciona* distal regeneration.

**Figure 8:**
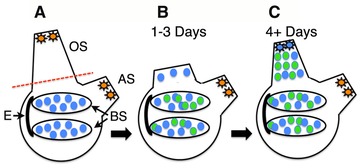
A diagrammatic illustration of temporal differences in the appearance of non‐proliferating (blue) and proliferating (green) branchial sac stem cells in the distal regeneration blastema. (A) An unoperated animal with non‐proliferating stem cells localized in the transverse vessels of the branchial sac. (B) Regenerating animal 1−3 h post‐amputation with non‐proliferating and proliferating stem cells in the transverse vessels and non‐proliferating stem cells in the distal regeneration blastema. (C) Regenerating animal 4+ days post‐amputation with non‐proliferating and proliferating stem cells in the transverse vessels and in the distal regeneration blastema. Some of the non‐proliferating stem cells have differentiated into siphon pigment organs. Dashed red line, site of amputation; star‐like structures, oral and atrial siphon pigment organs; OS, oral siphon; AS, atrial siphon; BS, branchial sac transverse vessels; E, endostyle.

A puzzling aspect of distal regeneration has been the delayed appearance of proliferating cells in the regeneration blastema (Auger et al. 2010). Cells that divide following distal extirpation are not evident in the regenerating region for about 4 days, although the wounded epidermis is repaired within a day (Sutton 1953) and structural components such as circular muscle bands and OPOs are replaced by about 4 days after the injury (Auger et al. 2010). The proliferating cells might already be present around the wound and delay their multiplication until about 4 days after the beginning of regeneration or they could initially divide somewhere else in the animal and subsequently migrate into the regeneration blastema. The present investigation has resolved this issue: short EdU labeling periods showed that the only site of intense cell proliferation relevant to distal regeneration is the vasculature and lymph nodes of the branchial sac, and pulse‐chase studies suggested that some of these cells subsequently migrate distally into the regeneration blastema.

Two lines of additional evidence support this conclusion. First, transplantation experiments showed that EdU labeled cells are able to migrate from the donor branchial sacs into distal regenerating areas of host animals. The pathway(s) taken by cells homing to the blastema remain unknown: they could move through the vasculature, they could migrate through tissue spaces and sinuses, or they could use both types of channels. Second, explants of distal oral siphon tissues did not contain substantial numbers of proliferating cells, although it is known that OPO regeneration occurs in these cultures (Auger et al. 2010). Thus, proliferating cells that originate from the branchial sac must be the precursors to most of the dividing cells in the regeneration blastema. Once these cells enter the regenerating area it seems likely that they continue to multiply and provide the basis for subsequent growth and differentiation after the initial re‐patterning events are completed.

### Branchial sac stem cells

The major source of proliferating cells that eventually migrate into the blastema are AP and PIWI expressing stem cells located in the lymph nodes and the transverse vessels of the branchial sac. Furthermore, evidence was obtained that the AP stained branchial sac stem cells themselves migrate into the sites of distal regeneration and differentiate directly into orange pigment cells in the OPOs. Masses of AP stained cells appear quickly in the regeneration blastema after distal extirpation without undergoing prior cell division, but are absent from siphon explants that regenerate OPOs in culture. It was previously shown that the oral siphon regeneration has a degree of autonomy in employing local sources of progenitor cells, providing that an injury is sustained in the most distal part of the structure (Auger et al. 2010). The small stem cell aggregations already present in the oral siphon, which have been detected for the first time in this study by AP staining, are likely to provide the precursors of OPOs that are reformed following such a distal injury. In contrast, when the injury is sustained near the base of the oral siphon or more proximal in the pharyngeal region, a massive invasion of stem cells from the branchial sac probably plays a major role in regeneration, and OPOs are replaced from outside the blastema by these precursors. A subset of these precursors may also be deposited in the distal areas to replenish the small local aggregations of AP positive stem cells.

The results of the present study suggest that the lymph nodes of the branchial sac and pharyngeal wall, which have been recognized previously as sources of renewing blood cells (Ermak 1976b; De Leo et al. 1987), are also responsible for the formation of new distal tissues and organs, including the siphons and potentially the neural complex. It was previously shown that non‐proliferating cells make up at least a part of the regenerating neural complex, but their source had not been evident (Bollner et al. 1997). Ascidians have many different types of blood cells (reviewed in Satoh 1994), including the hemoblasts: lymphocyte‐like cells that are responsible for producing all types of blood cells, as well as other somatic cells. For example, in colonial ascidians it has been shown that new muscle cells are derived from the hemoblasts during budding (Berrill 1941; Sugino et al. 2007). Further studies will be necessary to determine the identity of the *Ciona* blood cell types that invade the regeneration blastema with or without prior cell division. It seems likely, however, that these cells correspond to the lymphocyte‐like stem cells and one or more of their derivatives.

In colonial ascidians, stem cells producing precursors for growth and tissue replacement during budding and regeneration are localized in the endostyle and so‐called cell islands associated with this organ (Voskoboynik et al. 2008; Rinkevitch et al. 2013). In the case of distal regeneration in *Ciona*, however, the endostyle seems to be an unlikely candidate for a primary stem cell niche involved in distal regeneration because it is not labeled during a short EdU pulse after distal injury. Furthermore, although the *Ciona* endostyle contains PIWI positive cells, as described previously in a colonial ascidian (Brown et al. 2009), it does not contain AP positive cells. Because the endostyle was included in the donor branchial sacs used in our transplantation experiments, however, the possibility that this organ could also contribute cells to the distal regeneration blastema cannot be excluded with certainty.

Despite its inability to replace missing basal organs, distal regeneration via branchial sac vasculature in the solitary ascidian *Ciona* in some ways resembles whole body regeneration in botryllid colonial ascidians. During whole body regeneration somatic and germ tissues are replaced by mesenchymal stem cells and epidermis localized in the basal vasculature and ampullae that are activated after complete removal of zooids and buds from a colony (Rinkevich et al. 1995; Tiozzo et al. 2008; Brown et al. 2009). In contrast to colonial ascidians, adult *Ciona* do not exhibit an extensive system of basal vasculature; most of the adult vascular elements are confined to the branchial sac. In young adults, however, a vascular system is evident in the basal stalk. Interestingly, the basal stalk of *Ciona* also contains AP and PIWI positive stem cells but their roles in growth and regeneration, if any, remain to be elucidated.

### Age‐related depletion of branchial sac stem cells

Distal regeneration capacity, including reformation of the neural complex and oral siphon, has an inverse relationship with increased size in wild *Ciona* populations (Hirschler 1914; Dahlberg et al. 2009; Auger et al. 2010). Using size as a proxy for age it has been demonstrated that the capacity for distal regeneration declines and eventually ceases during aging in nature (Jeffery 2012). The present investigation has substantiated this conclusion in animals of precisely known age grown from fertilized eggs in a laboratory culture system. Most animals that have grown to old age in this system, and appear to be senile based on several criteria, were unable to regenerate oral siphons after extirpation, in contrast to the potent distal regeneration capacities of younger animals grown in the same system. Although the old animals retain proliferating cells in their branchial sacs, the structure of the latter are disorganized and the transverse vessels show reduced numbers of AP and PIWI stained stem cells relative to young animals. Moreover, the few old animals that retain some distal regenerative capacity had more AP stained stem cells in their branchial sacs than those unable to regenerate. Senile animals with irregular branchial sacs containing degenerate stigmata and transverse vessels have also been noted by Millar (1953) in wild *Ciona* populations.

Based on the results described above, we hypothesize that the decline in distal regeneration potential during aging may be caused by the depletion of a reservoir of branchial sac stem cells. This depletion could explain why OPOs are not replaced with fidelity after consecutive cycles of oral siphon amplification and regeneration in old animals (Jeffery 2012), as is possible in young animals (Auger et al. 2010). An attractive model for the relationship between the branchial sac stem cells and aging is that young animals may be endowed with most of the branchial sac stem cells needed for growth and regeneration throughout life, and when these cells are exhausted during old age the capacity for growth and replacement of distal structures is compromised.

### Stem cells in *Ciona* and vertebrate regeneration

The results of this investigation show that stem cells are important in *Ciona* distal regeneration. Stem cells are also important contributors to new tissue for regeneration of vertebrate body parts, such as the appendages (Poss et al. 2012). In *Ciona* most of the stem cells involved in distal regeneration arise from a distant source, the branchial sac, whereas in the regenerating vertebrate limb the stem cell source appears to be local. However, following amputation within the oral siphon itself, a local source of stem cells can also be used in *Ciona* regeneration (Auger et al. 2010). Thus, the differences in stem cell origin between *Ciona* distal regeneration and vertebrate limb regeneration may be minimal and dependent on the site of amputation. Furthermore, as shown here in *Ciona*, vertebrate stem (satellite) cells show reduced capacities for replacing skeletal muscle during aging (Conboy & Rando 2005; Gopinath & Rando 2008). These considerations suggest that the basic mechanisms of regeneration may be conserved among chordates. The relative simplicity of *Ciona* and other invertebrate chordates should make them useful models for helping to understand the complexity of vertebrate regeneration.

## Materials and Methods

### Animals and laboratory culture

Laboratory cultures were established using *Ciona intestinalis* (Type B) collected from the Cape Cod Canal near Woods Hole, MA, USA. Sperm and eggs were dissected from the gonoducts and *in vitro* fertilization was carried out in plastic Petri dishes containing Millipore filtered seawater (MFSW) using the gametes of two or more individuals. Zygotes were cultured at 18°C for 4−5 days post‐fertilization until metamorphosis was complete. The Petri dishes containing the post‐metamorphic juveniles were loaded onto racks in a laboratory culture system modeled after those described previously (Cirino et al. 2002; Joly et al. 2007). Some of the wild collected animals were used as hosts in branchial sac transplantation or explant cultures (see below). Unless stated otherwise, all experiments were conducted on approximately 3−4‐month‐old adults growing in culture on Petri dishes.

### Surgical operations

Animals were anesthetized in 0.2 mg/mL tricaine methanesulfonate (MS222) during the operations. Operations on large wild collected animals (3−10 cm in length) were conducted as described previously (Auger et al. 2010), and they were maintained in natural running seawater at ambient temperature during regeneration. *Ciona* are sensitive to surgical procedures that remove large parts of the bodies, which can evoke a stress response earmarked by a large increase in the number of orange pigment cells throughout the body and particularly the pharyngeal region (Parrinello et al. 2010). The stress response could occur even when animals were carefully removed from their normal attachments in nature or laboratory culture. To minimize this response, whenever possible the operations were carried out while animals were attached to Petri dishes, which also increased the survival rate. Operations were carried out using sharp tungsten needles, fine forceps, and straight bladed sharp microcautery scissors (Fine Science Tools Inc., Foster City, CA, USA).

### EdU labeling

Cell proliferation was detected by incorporation of 5‐ethynyl‐2′‐deoxyuridine (EdU) into replicating DNA. For pulse labeling experiments animals were incubated with 200 μmol/L EdU (Invitrogen, Carlsbad, CA, USA) in 100 mL of MFSW for 3−48 h while attached to Petri dishes. For chase experiments, the Petri dishes were rinsed three times with MFSW and incubated in 100 mL of MFSW or returned to the laboratory culture system. After completion of the labeling or chase, animals were fixed for 14 h in 4% formaldehyde, rinsed three times in 1 × phosphate‐buffered saline (PBS), permeabilized for 1 h with 0.5% Triton X‐100 in PBS, rinsed three times in PBS, and processed for EdU detection using Alexa Fluor azide 488 at room temperature according to the directions supplied with the Click‐iT™ EdU Alexa Fluor High Throughput Imaging Assay Kit (Invitrogen). Some of the EdU specimens were post‐fixed with 4% formaldehyde, embedded, and sectioned for histology as described below. The EdU labeled whole‐mount and sectioned specimens were imaged by fluorescence microscopy.

### Cell division inhibitor experiments

Inhibitor experiments were carried out at concentrations of 125 μg/mL colchicine or 0.05 μg/mL nocodazole (both purchased from Sigma‐Aldrich, St Louis, MO, USA) dissolved in MFSW. The optimal inhibitor concentrations were established by determining the effects on first cleavage during embryonic development. Some of the inhibitor experiments included a 24 h exposure to 200 μmol/L EdU followed by a 4 day chase to determine the effects on cell division.

### Alkaline phosphatase staining

For detection of stem cells by AP, small animals grown on culture plates were turned inside out as they were dissected from their tunics, which more readily exposed the internal tissues for reaction. This was accomplished by pressing on the basal area with a blunt forceps while the distal area was held firm with a sharp forceps and gradually pushing and pulling the stomach and intestine out of the tunic through the oral siphon aperture, which usually resulted in the entire animal turning inside out. Large animals were cut open to expose the internal tissues for AP staining or in some cases branchial sacs were stained after removal by dissection. The animals were fixed in 4% formaldehyde for 1 h at room temperature, washed three times in PBS, and then treated with BCIP‐NRH (Invitrogen) for 15 (small animals) or 60 (large animals) min at room temperature in the dark. Large animals were permeabilized prior to staining by treatment with 0.5% Triton‐100 in PBS. Controls were exposed to 100 μg/mL levamisole (Sigma‐Aldrich) for 1 h prior to staining. After stain development, the animals were washed three times in PBS and imaged.

### Protein extraction and PIWI western analysis

Proteins were extracted by homogenizing animals in RIPA buffer supplemented with 10% protease inhibitor cocktail (Sigma‐Aldrich). The homogenates were cleared by centrifugation, and the supernatants were subjected to electrophoresis through 10% sodium dodecyl sulfate/polyacrylamide (SDS/PAGE) gels containing pre‐stained protein markers and transferred to polyvinylidene fluoride filters (Bio‐Rad Laboratories, Hercules, CA, USA) (Harlow & Lane 1988). The filters were incubated with 5% non‐fat dry milk in TBST buffer (50 mmol/L Tris/HCl, pH 7.6, 100 mmol/L NaCl, 0.1% Tween‐20) to block non‐specific binding, washed three times for 5 min in TBST, incubated overnight in a 1:100 dilution of PIWI polyclonal antibody (ab5207, Abcam, Cambridge, MA, USA) in TBST at 4°C, and finally incubated for 1 h at room temperature in a 1:25000 dilution of horseradish‐peroxidase‐conjugated anti‐rabbit IgG secondary antibody. After three washes for 5 min with TBST, the signals were visualized with Chemiluminescence Luminol (Santa Cruz Biotechnology, Santa Cruz, CA, USA) used according to the supplier's specifications. Images of X‐ray films were taken with a digital camera and scanned, and band density was calculated by comparison with a glyceraldehyde‐3‐phosphate dehydrogenase (GAPDH) gel loading standard (Sigma‐Aldrich).

### PIWI immunostaining

Immunostaining used standard procedures (Jeffery et al. 2008) with PIWI antibody (see above). Animals were fixed in 4% paraformaldehyde in PBS (pH 7.0; overnight), stained with rabbit PIWI antibody diluted 1:100 in PBS, and antigen−antibody complexes were detected with biotinylated anti‐rabbit IgG secondary antibody (1:200 in PBS; Vector Laboratories, Burlingame, CA, USA) using the ABC Peroxidase Kit (Vector Laboratories). In control experiments, some animals were stained with a 1:100 dilution of rabbit polyclonal OCA2 antibody (70R‐6977; Fitzgerald Industries, Acton, MA, USA), which does not cross‐react with *Ciona*, and detected using the secondary antibody described above. Some of the stained specimens were post‐fixed with 4% paraformaldehyde overnight at 4°C, embedded and sectioned for histology as described below. The whole mounts and sections were viewed by light microscopy.

### Histological procedures

After post‐fixation (see above) the EdU labeled and AP or PIWI stained specimens were dehydrated to 100% ethanol, embedded in polyester wax (Norenburg & Barrett 1987), and sectioned at 10 μm. The sections were placed on glass slides, de‐embedded, and imaged by fluorescence or bright field microscopy.

### Branchial sac transplantations

For branchial sac transplantations, the donor animals were first labeled with EdU for 48 h as described above and then dyed with 0.1% Neutral Red. The branchial sac complex, which included the entire branchial sac, endostyle, and associated vasculature, was dissected away from the viscera and other tissues and transplanted into the distal branchial area below the oral siphon of hosts. The donor transplant was inserted into a small lesion made in the host branchial sac through a window cut into the tunic. After transplantation, the window was sealed by covering with adjacent tunic, the host was removed from anesthesia to a small volume of MFSW, which induced contraction and wound closing, and fusion of donor and host tissues was promoted by incubation for 12 h at room temperature. If the transplant was rejected during this period, which sometimes occurred by discharging the donor transplant through the tunic window or into the pharynx with subsequent expulsion through the oral siphon, the transplantation process was repeated with the same donor and host. The transplantation was repeated as many times as needed to obtain stable incorporation into the host. Subsequently, the host was removed to a large tank of circulating natural seawater at ambient temperature for recovery. After about 4−5 days of recovery, the oral siphon of the host animal was amputated as described above, and the host animal was subsequently returned to the tank of circulating seawater for regeneration. After about 10−15 days, the distal halves of regenerating hosts were excised while under anesthesia and subjected to EdU detection procedures as described above. EdU incorporation was quantified by counting labeled cells in flat mounts of hosts prepared as described previously (Jeffery 2012).

### Explant cultures

The preparation and *in vitro* culture of explants was carried out at room temperature in 10% Gibco Neurobasal Medium (Invitrogen) in MFSW containing antibiotics as described previously (Auger et al. 2010) except that the culture medium was supplemented with 200 μmol/L EdU, media was changed twice daily, and after 5 days the cultures were assayed for EdU detection and AP staining as described above.
